# Risk of Parkinson disease according to metabolic syndrome among Korean adults: an analysis of data from the National Health Insurance–National Sample Cohort

**DOI:** 10.4178/epih.e2026024

**Published:** 2026-06-03

**Authors:** Je-Hyun Kim, Hae-Sung Nam, Ju-Mi Lee

**Affiliations:** 1 Department of Public Health, Graduate School, Chungnam National University, Daejeon, Korea; 2 Department of Preventive Medicine, Chungnam National University College of Medicine, Daejeon, Korea

**Keywords:** Metabolic syndrome, Parkinson disease, Cohort studies

## Abstract

**OBJECTIVES:**

This study investigated the association of metabolic syndrome (MetS) and its components with the risk of developing Parkinson disease (PD) among Korean adults using the National Health Insurance Service–National Sample Cohort (NHIS-NSC).

**METHODS:**

We analyzed 363,602 adults aged ≥40 years without a prior diagnosis of PD who underwent national health examinations between 2010 and 2014, using data from the NHIS-NSC version 2.2 database. Participants were categorized into MetS and non-MetS groups and were followed from the baseline examination until PD diagnosis, death, or December 31, 2019. Cox proportional hazards models were used to estimate hazard ratios (HRs) and 95% confidence intervals (CIs), with sequential adjustment for covariates.

**RESULTS:**

During a mean follow-up of 8.2 years, 2,834 incident PD cases were identified. In the fully adjusted Cox model, MetS was associated with a significantly higher risk of developing PD (HR, 1.22; 95% CI, 1.13 to 1.31). Among the individual MetS components, abdominal obesity (HR, 1.19; 95% CI, 1.10 to 1.29) and elevated fasting blood glucose (HR, 1.20; 95% CI, 1.11 to 1.29) were significantly associated with an increased risk of PD. PD incidence also increased as the number of MetS components increased (log-rank p<0.001).

**CONCLUSIONS:**

These findings suggest that MetS and its components, particularly abdominal obesity and hyperglycemia, are associated with an increased risk of PD. Effective management of MetS may therefore help reduce the future burden of PD.

## GRAPHICAL ABSTRACT

[Fig f3-epih-48-e2026024]

## Key Message

This retrospective cohort study of Korean adults aged 40 and older showed that metabolic syndrome and its components, particularly abdominal obesity and hyperglycemia, increased the incidence of Parkinson disease. Effective management of metabolic syndrome may help reduce the future burden of Parkinson disease.

## INTRODUCTION

Parkinson disease (PD) is the second most common neurodegenerative disease among individuals aged ≥65 years. The disease is characterized by motor symptoms such as bradykinesia, resting tremor, rigidity, and gait or postural instability, which arise from progressive loss of dopaminergic neurons in the substantia nigra [1,2]. Although the incidence and prevalence of PD are increasing worldwide because of population aging, the mechanisms underlying PD-related neurodegeneration remain incompletely understood. Experimental studies using animal and cell culture models have suggested that increased oxidative stress may impair neuronal energy metabolism in PD [3].

Metabolic syndrome (MetS) is diagnosed when at least 3 of the following components are present: abdominal obesity, elevated triglycerides, reduced high-density lipoprotein (HDL) cholesterol, elevated blood pressure, and elevated fasting blood glucose (FBG) [4]. MetS affects more than one-fifth of adults in many countries, including Korea, and its rising prevalence has made it a major global public health concern [5]. It is also a well-established risk factor for atherosclerotic and non-atherosclerotic cardiovascular and cerebrovascular diseases [6].

MetS has been proposed to contribute to PD risk through several pathophysiological mechanisms, including insulin resistance, chronic inflammation, and oxidative stress [2,6]. However, epidemiological evidence supporting this association remains limited [7-10]. Korean and Chinese cohort studies [7-9] reported an increased risk of PD associated with MetS, but their follow-up periods were relatively short. In contrast, a Finnish cohort study [10] based on a relatively small baseline sample reported an inverse association between MetS and PD.

Given these limitations in the existing evidence, we investigated the association of MetS and its components with the risk of developing PD among Korean adults. We used the National Health Insurance Service–National Sample Cohort (NHIS-NSC) version 2.2 database, which includes follow-up data through 2019.

## MATERIALS AND METHODS

### Data

This study used data from the NHIS-NSC version 2.2 database (NHIS approval No. NHIS-2024-2-174). The NHIS-NSC is a large population-based cohort that includes approximately 1 million individuals and represents approximately 2.2% of the general Korean population. The NHIS provides biennial health examinations comprising a health survey, physical examination, and laboratory testing [11]. In the present study, 536,197 participants had a unique baseline health examination record between 2010 and 2014, representing roughly 54% of the approximately 1 million-member cohort.

**Figure 1.**
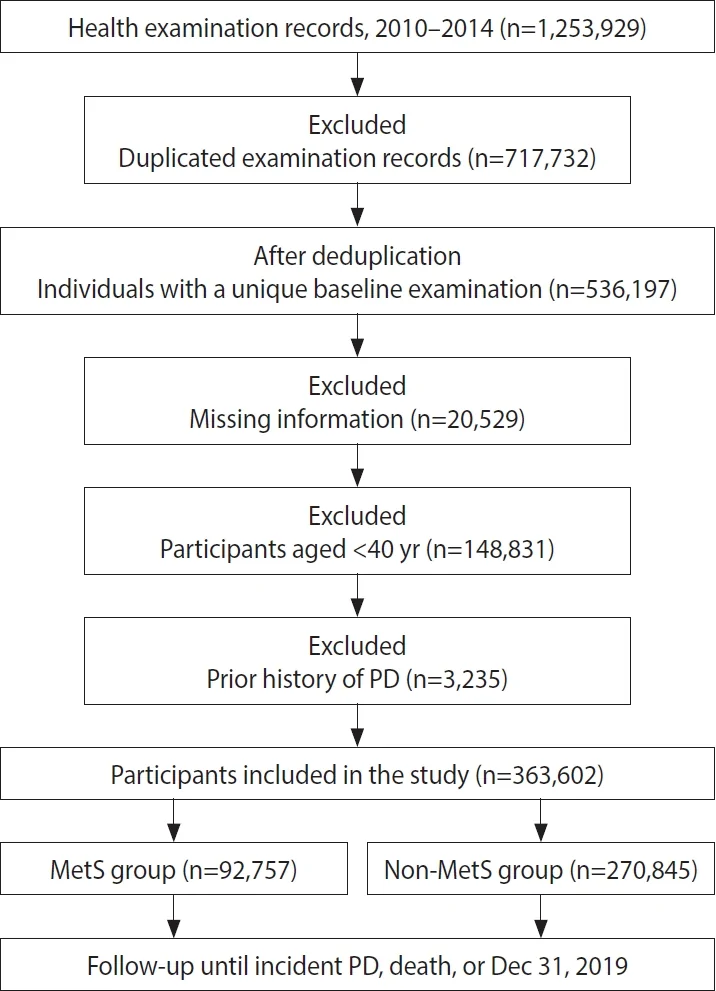
Flow chart of participant selection and classification from the National Health Insurance Service–National Sample Cohort version 2.2. PD, Parkinson disease; MetS, metabolic syndrome.

We initially identified 1,253,929 NHIS health screening examination records collected between 2010 and 2014. Records were deduplicated at the individual level, and each participant’s earliest examination date was defined as baseline, yielding 536,197 individuals with a unique baseline examination. We then excluded participants with missing data required to define MetS or ascertain covariates included in the fully adjusted model, those aged <40 years, and those with a prior diagnosis of PD, defined as any record of International Classification of Diseases, 10th revision (ICD-10) code G20 before the baseline health examination. The final analytic cohort comprised 363,602 participants (Figure 1), including 92,757 individuals with MetS and 270,845 without MetS. Participants were followed until PD onset, death, or December 31, 2019.

### Baseline measurements: metabolic syndrome and covariates

Baseline measurements were obtained from NHIS health examinations conducted according to standardized procedures across participating institutions. Height, weight, and waist circumference were measured, and body mass index (BMI) was calculated as weight in kilograms divided by height in meters squared (kg/m^2^). Systolic blood pressure (SBP) and diastolic blood pressure (DBP) were measured in the sitting position after at least 5 minutes of rest. After an 8-hour fast, blood samples were collected to measure FBG, total cholesterol, triglycerides, HDL cholesterol, and low-density lipoprotein cholesterol.

MetS was defined as the presence of 3 or more of the following 5 components according to the harmonized criteria for metabolic syndrome [12]. For abdominal obesity, Asian-specific waist circumference cutoffs recommended by the Korean Society of Obesity were applied [13]. The 5 components were defined as follows: (1) abdominal obesity: waist circumference ≥90 cm in male and ≥85 cm in female; (2) elevated triglycerides: triglyceride level ≥150 mg/dL or current use of medication for hypertriglyceridemia; (3) low HDL cholesterol: HDL cholesterol <40 mg/dL in male or <50 mg/dL in female; (4) elevated blood pressure: SBP ≥130 mmHg or DBP ≥85 mmHg, or current use of antihypertensive medication; (5) elevated FBG: FBG ≥100 mg/dL or current use of antidiabetic medication.

Smoking status, alcohol consumption, and physical activity were assessed using standardized self-report questionnaires administered during the NHIS health examinations. Current and former smokers were classified as smokers, and participants who consumed alcohol at least once per week were classified as drinkers. Participants who reported walking for at least 30 minutes per week were classified as physically active. Income level was dichotomized by classifying individuals in the lowest 20% of insurance premiums as the low-income group. Baseline comorbidities were identified from NHIS medical utilization records when the relevant ICD-10 codes appeared as primary or secondary diagnoses during the 1-year look-back period before baseline: I10–I15 for hypertension, E10–E14 for diabetes mellitus, E78 for dyslipidemia, and I20–I25 for ischemic heart disease.

### Follow-up observations for the onset of Parkinson disease

Incident PD was defined as the first claim with ICD-10 code G20 recorded as a primary or secondary diagnosis in the NHIS claims database, consistent with previous claims-based studies [7,8]. To improve diagnostic specificity, participants whose G20 code was recorded as an “excluded diagnosis” during follow-up were not classified as incident PD cases. Because this additional exclusion has not been formally validated, we performed a sensitivity analysis using a stricter PD definition that additionally required registration under the Rare and Intractable Diseases program (V124).

### Statistical analysis

Statistical analyses were conducted using SAS version 9.4 (SAS Institute Inc., Cary, NC, USA). The PD incidence rate was calculated as the number of incident PD cases per 1,000 person-years. Baseline characteristics were compared according to MetS status using the independent-samples t-test for continuous variables and the chi-square test for categorical variables.

Cox proportional hazards models were used to estimate hazard ratios (HRs) and 95% confidence intervals (CIs) for incident PD according to MetS status, individual MetS components, and the number of MetS components. Model 1 was unadjusted. Model 2 was adjusted for age and sex. Model 3 was additionally adjusted for income level, smoking status, alcohol consumption, physical activity, and BMI; age and BMI were modeled as continuous variables.

Sensitivity analyses were performed as follows: (1) exclusion of participants with pre-baseline ischemic stroke (ICD-10 codes I63–I64), exclusion of PD cases occurring within the first 5 years of follow-up, and simultaneous application of both exclusions; (2) additional adjustment for baseline comorbidities, including hypertension, diabetes mellitus, dyslipidemia, and ischemic heart disease, which may be influenced by MetS or lie on the causal pathway and therefore were not included in the primary model to avoid potential over adjustment [14]; (3) use of a stricter PD definition requiring registration under the Rare and Intractable Diseases program (V124) among cases identified using the primary definition; and (4) flexible modeling of age using restricted cubic splines. Subgroup analyses were also performed according to age group, 40–64 years and ≥65 years, and sex. Interaction tests were conducted to assess potential effect modification.

Kaplan–Meier survival curves were used to illustrate cumulative PD incidence according to the number of MetS components, and differences between groups were assessed using the log-rank test. A p-value <0.05 was considered statistically significant.

### Ethics statement

The institutional review board exempted this study from ethical review (IRB No. 202303-SB-041-01).

## RESULTS

### Baseline characteristics of study participants

Table 1 presents the baseline characteristics of participants according to MetS status. Compared with the non-MetS group, the MetS group included a higher proportion of male and had a higher mean age and BMI (all p<0.001).

### Incidence and hazard ratios for Parkinson disease according to metabolic syndrome status and components

During a mean follow-up of 8.2 years, 2,834 of the 363,602 participants developed incident PD. The PD incidence rate was higher in the MetS group than in the non-MetS group (1.45 vs. 0.79 per 1,000 person-years). In Model 3, which included adjustment for all covariates, participants with MetS had a 22% higher risk of developing PD than those without MetS (HR, 1.22; 95% CI, 1.13 to 1.31; Table 2). The association remained robust across sensitivity analyses. First, MetS remained significantly associated with PD after excluding participants with pre-baseline ischemic stroke (ICD-10 codes I63–I64; HR, 1.22; 95% CI, 1.12 to 1.32), after excluding PD cases diagnosed within the first 5 years of follow-up (HR, 1.17; 95% CI, 1.06 to 1.29), and after applying both exclusions simultaneously (HR, 1.16; 95% CI, 1.04 to 1.29). Second, additional adjustment for major comorbidities produced a slightly attenuated but still significant association (HR, 1.12; 95% CI, 1.03 to 1.21). Third, when PD cases identified by the primary definition were further restricted to those registered under the Rare and Intractable Diseases program (code V124), the HR was attenuated but remained statistically significant (HR, 1.14; 95% CI, 1.00 to 1.30; p=0.046). Finally, flexible modeling of age using restricted cubic splines did not materially change the association (HR, 1.20; 95% CI, 1.11 to 1.29).

In subgroup analyses, the association between MetS and PD was observed among both participants aged 40–64 years (HR, 1.21; 95% CI, 1.05 to 1.39) and those aged ≥65 years (HR, 1.18; 95% CI, 1.07 to 1.30), with no significant interaction by age group (p for interaction=0.61). Similarly, the association remained positive in both male (HR, 1.14; 95% CI, 1.00 to 1.29) and female (HR, 1.28; 95% CI, 1.16 to 1.43), with no evidence of interaction by sex (p for interaction=0.52).

Among the individual MetS components, abdominal obesity (HR, 1.19; 95% CI, 1.10 to 1.29) and elevated FBG (HR, 1.20; 95% CI, 1.11 to 1.29) were associated with an increased risk of PD. In contrast, elevated triglycerides, low HDL cholesterol, and elevated blood pressure were not statistically associated with PD risk.

### Hazard ratios for Parkinson disease according to the number of metabolic syndrome components

**Figure 2.**
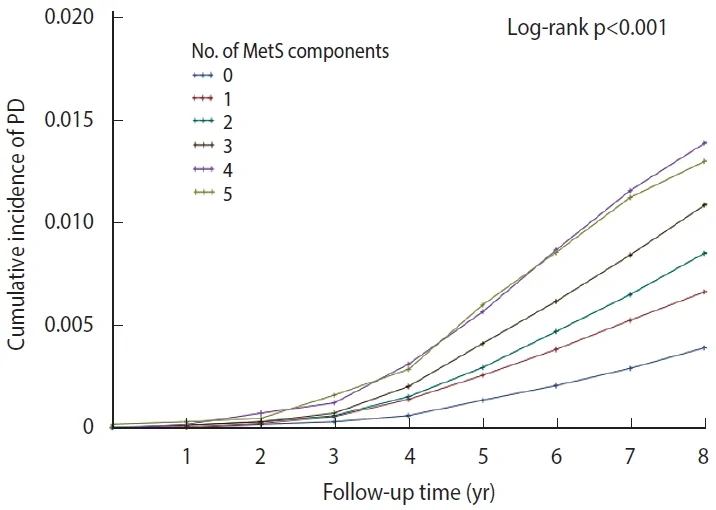
Cumulative incidence of Parkinson disease (PD) according to the number of metabolic syndrome (MetS) components.

Kaplan–Meier survival curves (Figure 2) indicated that cumulative PD incidence increased as the number of MetS components increased (log-rank p<0.001). Consistent with this unadjusted pattern, adjusted Cox proportional hazards models showed a higher risk of PD with increasing numbers of MetS components (Table 3). Compared with participants with no MetS components, those with 4 components had a 54% higher risk of PD (HR, 1.54; 95% CI, 1.32 to 1.80). Participants with all 5 components also showed an increased risk (HR, 1.20; 95% CI, 0.94 to 1.54), although this association was not statistically significant.

## DISCUSSION

During a mean follow-up of 8.2 years, 2,834 of 363,602 participants developed PD, corresponding to an overall incidence rate of 0.95 per 1,000 person-years. This incidence rate was comparable to previously reported rates among Korean adults aged ≥40 years [15], supporting the representativeness of the cohort.

We found that participants with MetS had a significantly higher risk of PD than those without MetS, even after adjustment for potential confounders (HR, 1.22; 95% CI, 1.13 to 1.31).

Although the HR was attenuated after adjustment for age and sex, the association remained materially unchanged when age was modeled flexibly using restricted cubic splines. Similar associations were also observed across age and sex strata, with no significant interaction. These findings suggest that the main result was not explained solely by age-model specification or by effect heterogeneity according to age or sex.

Compared with the previous nationwide Korean study [7], which had a mean follow-up duration of 5.3 years, the present study extended follow-up through 2019 and had a longer mean follow-up duration of 8.2 years. In addition, we performed several complementary analyses, including a more stringent 5-year lag analysis, a stricter PD definition requiring the Rare and Intractable Diseases registration code (V124), flexible modeling of age, and subgroup analyses according to age and sex. These analyses strengthened the robustness and interpretability of the observed association by helping address concerns regarding reverse causation, outcome misclassification, and model specification, although the association was attenuated under the stricter V124 definition.

The risk of PD increased progressively with the number of MetS components, suggesting a dose–response relationship consistent with findings from recent meta-analyses and large-scale cohort studies [16,17]. However, the HR for participants with all 5 MetS components was lower than that for participants with 4 components and was not statistically significant. This finding should be interpreted cautiously. First, the number of PD cases in the 5-component group was relatively small, which may have limited statistical precision. Second, survival-related selection cannot be excluded. Because MetS has been associated with increased all-cause mortality in previous meta-analyses [18], participants with the highest metabolic burden may have been more likely to die or experience other serious health events before PD was clinically recognized. In addition, loss to follow-up or survival-related selection may threaten the validity of effect estimates in cohort studies [19]. Therefore, the non-significant finding in the 5-component group should not be interpreted as evidence of no association.

Among the MetS components, abdominal obesity and elevated FBG were significantly associated with an increased risk of PD, whereas elevated triglycerides, low HDL cholesterol, and elevated blood pressure were not statistically significant. This pattern suggests that glucose metabolism and insulin resistance may play more direct roles in the neurodegenerative processes leading to PD [2,6]. Previous mechanistic studies have reported that impaired insulin signaling, mitochondrial dysfunction, and neuroinflammation may contribute to dopaminergic neuronal loss, providing biological plausibility for the observed association between MetS and PD [2,3,6]. Recent literature has also highlighted the potential relevance of glucagon-like peptide-1 (GLP-1)-related signaling in PD. GLP-1 is an incretin hormone involved in insulin secretion and metabolic regulation, and its receptor is expressed in brain regions relevant to PD, where receptor activation has been linked to modulation of neuroinflammation, mitochondrial function, and neuronal survival [20]. This evidence further supports the biological plausibility of stronger associations for abdominal obesity and hyperglycemia.

From a public health perspective, these findings highlight the importance of early identification and management of MetS in middle-aged and older adults. Lifestyle interventions, including weight control, dietary modification, and physical activity, may not only reduce cardiovascular and metabolic risks but also potentially attenuate the future burden of PD. Integrating metabolic health management into preventive neurology may therefore represent a novel approach to reducing PD incidence at the population level.

The limitations of this study should be considered. First, claims-based PD ascertainment may lead to outcome misclassification. To reduce misclassification, individuals whose G20 code later appeared as an excluded diagnosis during follow-up were excluded, thereby avoiding the inclusion of provisional or rule-out diagnoses as incident PD cases. Because this approach has not been formally validated, we additionally performed a sensitivity analysis using a stricter V124-based definition, which may improve specificity but reduce sensitivity because it reflects completion of an administrative registration process rather than the earliest clinical diagnosis. In that sensitivity analysis, the effect estimate was attenuated, but the direction of the association remained consistent with the main findings. Medication-based definitions may likewise miss early or mild cases because treatment initiation is individualized [21]. Second, reverse causality cannot be fully excluded despite the lagged analyses, given the long prodromal phase of PD. These limitations underscore the need for cautious interpretation of the findings. Moreover, the claims-based dataset did not include detailed information on certain lifestyle or environmental factors relevant to PD risk, including caffeine intake and pesticide exposure. Because these factors have been associated with PD risk in prior studies, residual confounding cannot be excluded [22,23].

Future research should elucidate the biological mechanisms linking MetS to PD, particularly those involving insulin signaling pathways, neuroinflammation, and mitochondrial dysfunction. Studies incorporating direct biomarkers of insulin resistance, a key underlying feature of MetS, may help clarify the mechanisms underlying the observed association between MetS and PD. Longitudinal studies, including intervention-oriented research, would also be valuable for determining whether improved management of MetS is associated with a lower future incidence of PD. Replication studies in non-Korean populations are needed to confirm the generalizability of these associations.

In conclusion, our findings suggest that MetS and its components, particularly abdominal obesity and hyperglycemia, are associated with an increased risk of PD. Effective management of MetS may therefore be important not only for cardiometabolic health but also for reducing the risk of neurodegenerative diseases such as PD.

## Figures and Tables

**Figure f3-epih-48-e2026024:**
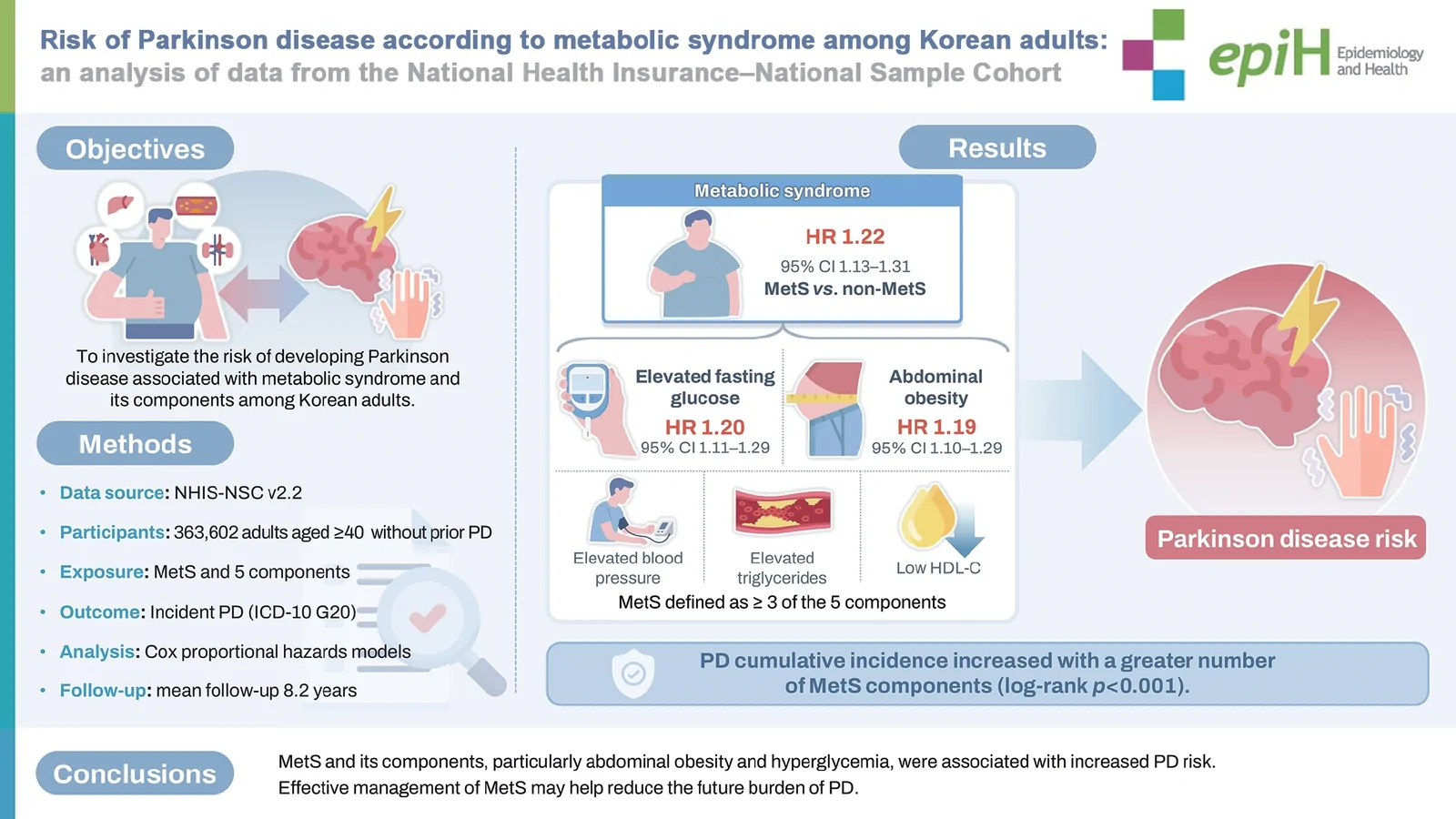


**Table 1.** Baseline of study participants according to MetS status

Characteristics	MetS (n=92,757)	Non-MetS (n=270,845)	p-value^1^
Age (yr)	58.3±11.2	53.3±10.5	<0.001
Male	48,471 (52.3)	121,186 (44.7)	<0.001
BMI (kg/m^2^)	26.1±10.8	23.2±2.8	<0.001
Waist circumference (cm)	87.8±8.1	78.6±7.9	<0.001
Systolic BP (mmHg)	132.6±14.8	120.8±14.6	<0.001
Diastolic BP (mmHg)	81.5±10.1	75.6±9.8	<0.001
FBG (mg/dL)	114.8±34.1	95.6±20.4	<0.001
Total cholesterol (mg/dL)	201.6±44.8	197.2±38.1	<0.001
HDL cholesterol (mg/dL)	47.8±21.7	58.0±25.0	<0.001
LDL cholesterol (mg/dL)	116.4±75.5	118.3±53.1	<0.001
Triglyceride (mg/dL)^2^	179.0 (132.0-237.0)	98.0 (71.0-133.0)	<0.001
Smoking status			<0.001
Yes	37,075 (40.0)	92,061 (34.0)	
No	55,682 (60.0)	178,784 (66.0)	
Alcohol drinker			<0.001
Yes	80,438 (86.7)	224,693 (82.9)	
No	12,319 (13.3)	46,152 (17.1)	
Physically active			<0.001
Yes	57,871 (62.4)	176,185 (65.1)	
No	34,886 (37.6)	94,660 (34.9)	
Low income (lower 20%)	14,431 (15.6)	42,655 (15.8)	0.017
Comorbidities			<0.001
Hypertension	47,797 (51.5)	51,024 (18.8)	
Diabetes mellitus	24,861 (26.8)	21,588 (8.0)	
Dyslipidemia	30,845 (33.3)	41,939 (15.5)	
Ischemic heart disease	8,577 (9.2)	11,121 (4.1)	

Values are presented as mean±standard deviation or number (%), unless otherwise indicated. MetS, metabolic syndrome; BMI, body mass index; BP, blood pressure; FBG, fasting blood glucose; HDL, high-density lipoprotein; LDL, low-density lipoprotein.

1 Using the Student t-test for continuous variable and the chi-square test for categorical variables.

2 Triglyceride levels are presented as median (interquartile range); p-values were calculated using the Wilcoxon rank sum test.

**Table 2.** Incidence and hazard ratios for PD according to MetS status and components^1^

Variables	PD	Person-years	IR^2^	Model 1	Model 2	Model 3
MetS						
No	1,743	2,218,213	0.79	1.00 (reference)	1.00 (reference)	1.00 (reference)
Yes	1,091	749,871	1.45	1.87 (1.73, 2.02)	1.22 (1.13, 1.32)	1.22 (1.13, 1.31)
Abdominal obesity^3^						
No	1,905	2,297,442	0.83	1.00 (reference)	1.00 (reference)	1.00 (reference)
Yes	929	670,642	1.39	1.68 (1.55, 1.82)	1.19 (1.10, 1.29)	1.19 (1.10, 1.29)
Elevated triglycerides^4^						
No	1,821	2,016,550	0.90	1.00 (reference)	1.00 (reference)	1.00 (reference)
Yes	1,013	951,534	1.06	1.18 (1.09, 1.27)	1.07 (0.99, 1.15)	1.06 (0.99, 1.15)
Low HDL cholesterol^5^						
No	2,012	2,286,366	0.88	1.00 (reference)	1.00 (reference)	1.00 (reference)
Yes	822	681,718	1.21	1.38 (1.27, 1.49)	1.08 (1.00, 1.18)	1.08 (1.00, 1.18)
Elevated blood pressure^6^						
No	967	1,525,446	0.63	1.00 (reference)	1.00 (reference)	1.00 (reference)
Yes	1,867	1,442,638	1.29	2.05 (1.90, 2.22)	1.08 (1.00, 1.18)	1.08 (1.00, 1.17)
Elevated FBG^7^						
No	1,469	1,865,521	0.79	1.00 (reference)	1.00 (reference)	1.00 (reference)
Yes	1,365	1,102,563	1.24	1.59 (1.48, 1.72)	1.20 (1.11, 1.29)	1.20 (1.11, 1.29)

Values are presented as hazard ratio (95% confidence interval); “No” indicates not meeting the corresponding criteria. PD, Parkinson disease; MetS, metabolic syndrome; IR, incidence rate; HDL, high-density lipoprotein; FBG, fasting blood glucose.

1 Model 1 was unadjusted; Model 2 was adjusted for age and sex; Model 3 was adjusted for age, sex, smoking status, alcohol consumption, physical activity, body mass index, and income.

2 PD incidence per 1,000 person-years.

3 Abdominal obesity (yes): waist circumference ≥90 cm in male or ≥85 cm in female.

4 Elevated triglycerides (yes): triglycerides ≥150 mg/dL or on medication for elevated triglycerides.

5 Low HDL cholesterol (yes): HDL cholesterol <40 mg/dL in male or <50 mg/dL in female.

6 Elevated blood pressure (yes): systolic blood pressure ≥130 mmHg or diastolic blood pressure ≥85 mmHg, or on antihypertensive medication.

7 Elevated FBG (yes): FBG ≥100 mg/dL or on antidiabetic medication.

**Table 3.** Hazard ratios for PD according to number of MetS components^1^

No. of MetS components	PD	Person-years	IR^2^	Model 1	Model 2	Model 3
0	315	666,430	0.47	1.00 (reference)	1.00 (reference)	1.00 (reference)
1	665	824,756	0.81	1.70 (1.49, 1.95)	1.15 (1.01, 1.32)	1.15 (1.01, 1.32)
2	763	727,027	1.05	2.23 (1.95, 2.54)	1.18 (1.03, 1.35)	1.18 (1.03, 1.35)
3	639	480,486	1.33	2.83 (2.47, 3.24)	1.33 (1.16, 1.52)	1.32 (1.15, 1.52)
4	372	218,098	1.71	3.66 (3.15, 4.25)	1.55 (1.33, 1.80)	1.54 (1.32, 1.80)
5	80	51,287	1.56	3.36 (2.63, 4.30)	1.21 (0.94, 1.55)	1.20 (0.94, 1.54)

Values are presented as hazard ratio (95% confidence interval). PD, Parkinson disease; MetS, metabolic syndrome; IR, incidence rate.

1 Model 1 was unadjusted; Model 2 was adjusted for age and sex; Model 3 was adjusted for age, sex, smoking status, alcohol consumption, physical activity, body mass index, and income.

2 PD incidence per 1,000 person-years.

## References

[1] Poewe W, Seppi K, Tanner CM, Halliday GM, Brundin P, Volkmann J (2017). Parkinson disease. Nat Rev Dis Primers.

[2] Li LY, Liu SF, Zhuang JL, Li MM, Huang ZP, Chen YH (2022). Recent research progress on metabolic syndrome and risk of Parkinson’s disease. Rev Neurosci.

[3] Mattson MP, Pedersen WA, Duan W, Culmsee C, Camandola S (1999). Cellular and molecular mechanisms underlying perturbed energy metabolism and neuronal degeneration in Alzheimer’s and Parkinson’s diseases. Ann N Y Acad Sci.

[4] Alberti KG, Zimmet P, Shaw J; IDF Epidemiology Task Force Consensus Group (2005). The metabolic syndrome--a new worldwide definition. Lancet.

[5] Ranasinghe P, Mathangasinghe Y, Jayawardena R, Hills AP, Misra A (2017). Prevalence and trends of metabolic syndrome among adults in the Asia-Pacific region: a systematic review. BMC Public Health.

[6] Zhang P, Tian B (2014). Metabolic syndrome: an important risk factor for Parkinson’s disease. Oxid Med Cell Longev.

[7] Nam GE, Kim SM, Han K, Kim NH, Chung HS, Kim JW (2018). Metabolic syndrome and risk of Parkinson disease: a nationwide cohort study. PLoS Med.

[8] Roh JH, Lee S, Yoon JH (2021). Metabolic syndrome and Parkinson’s disease incidence: a nationwide study using propensity score matching. Metab Syndr Relat Disord.

[9] Peng Z, Zhou R, Liu D, Cui M, Yu K, Yang H (2021). Association between metabolic syndrome and mild Parkinsonian signs progression in the elderly. Front Aging Neurosci.

[10] Sääksjärvi K, Knekt P, Männistö S, Lyytinen J, Heliövaara M (2015). Prospective study on the components of metabolic syndrome and the incidence of Parkinson’s disease. Parkinsonism Relat Disord.

[11] Lee J, Lee JS, Park SH, Shin SA, Kim K (2017). Cohort profile: the National Health Insurance Service-National Sample Cohort (NHIS-NSC), South Korea. Int J Epidemiol.

[12] Alberti KG, Eckel RH, Grundy SM, Zimmet PZ, Cleeman JI, Donato KA (2009). Harmonizing the metabolic syndrome: a joint interim statement of the International Diabetes Federation Task Force on Epidemiology and Prevention; National Heart, Lung, and Blood Institute; American Heart Association; World Heart Federation; International Atherosclerosis Society; and International Association for the Study of Obesity. Circulation.

[13] Haam JH, Kim BT, Kim EM, Kwon H, Kang JH, Park JH (2023). Diagnosis of obesity: 2022 update of clinical practice guidelines for obesity by the Korean Society for the Study of Obesity. J Obes Metab Syndr.

[14] Schisterman EF, Cole SR, Platt RW (2009). Overadjustment bias and unnecessary adjustment in epidemiologic studies. Epidemiology.

[15] Kang SH, Moon SJ, Kang M, Chung SJ, Cho GJ, Koh SB (2023). Incidence of Parkinson’s disease and modifiable risk factors in Korean population: a longitudinal follow-up study of a nationwide cohort. Front Aging Neurosci.

[16] Zhong Y, Wang TH, Huang LJ, Hua YS (2024). Association between metabolic syndrome and the risk of Parkinson’s disease: a meta-analysis. BMC Neurol.

[17] Zhang X, Wang J, Dove A, Yu T, Li Q, Gottesman RF (2025). Metabolic syndrome and incidence of Parkinson disease: a community-based longitudinal study and meta-analysis. Neurology.

[18] Mottillo S, Filion KB, Genest J, Joseph L, Pilote L, Poirier P (2010). The metabolic syndrome and cardiovascular risk a systematic review and meta-analysis. J Am Coll Cardiol.

[19] Howe CJ, Cole SR, Lau B, Napravnik S, Eron JJ (2016). Selection bias due to loss to follow up in cohort studies. Epidemiology.

[20] Orozco MP, Vintimilla Rivadeneira V, Leon-Rojas JE (2025). GLP-1 signalling as a therapeutic avenue in Parkinson’s disease: a comprehensive review. Int J Mol Sci.

[21] Lee JJ (2019). Pharmacological treatment in Parkinson’s disease. J Korean Neurol Assoc.

[22] Costa J, Lunet N, Santos C, Santos J, Vaz-Carneiro A (2010). Caffeine exposure and the risk of Parkinson’s disease: a systematic review and meta-analysis of observational studies. J Alzheimers Dis.

[23] van der Mark M, Brouwer M, Kromhout H, Nijssen P, Huss A, Vermeulen R (2012). Is pesticide use related to Parkinson disease? Some clues to heterogeneity in study results. Environ Health Perspect.

